# Vascular risk factors, white matter lesions and cognitive impairment in Parkinson’s disease: the PACOS longitudinal study

**DOI:** 10.1007/s00415-020-10189-8

**Published:** 2020-08-31

**Authors:** Alessandra Nicoletti, Antonina Luca, Roberta Baschi, Calogero Edoardo Cicero, Giovanni Mostile, Marco Davì, Giuseppe La Bianca, Vincenzo Restivo, Mario Zappia, Roberto Monastero

**Affiliations:** 1 Department of Medical, Surgical Sciences and Advanced Technologies, GF Ingrassia, University of Catania, Catania, Italy; 2 Department of Biomedicine, Neuroscience and Advanced Diagnostics, University of Palermo, Palermo, Italy; 3 Department of Sciences for Health Promotion and Mother-Child Care, University of Palermo, Palermo, Italy

**Keywords:** Parkinson’s disease, Mild cognitive impairment, Hypertension, White matter lesions, Risk factors, Epidemiology

## Abstract

**Background:**

Vascular risk factors (VRFs) may be associated with cognitive decline in early Parkinson’s disease (PD) but results are inconclusive. The identification of modifiable risk factors is relevant for prevention and treatment.

**Methods:**

Parkinson’s disease (PD) patients of the PACOS cohort who underwent a baseline and follow-up neuropsychological evaluation were enrolled in the study. PD with Mild Cognitive Impairment (MCI) and dementia (PDD) were diagnosed according to the MDS criteria. A Baseline 1.5 T brain MRI was used to calculate the white matter lesions (WMLs) burden using the Wahlund visual scale. Laboratory data, presence of hypertension, diabetes and use of anti-hypertensive drugs were collected and the Framingham Risk (FR) score was calculated. VRFs predicting PD-MCI and PDD were evaluated using Cox proportional hazard regression model.

**Results:**

Out of 139 enrolled patients, 84 (60.4%) were classified as normal cognition (NC) and 55 (39.6%) as MCI at baseline. At follow-up 28 (33.3%) PD-NC developed MCI and 4 (4.8%) PDD (follow-up time 23.5 ± 10.3 months). Out of 55 PD-MCI patients at baseline, 14 (25.4%) converted to PDD. At multivariate analysis among PD-NC a systolic blood pressure (SBP) > 140 mmHg was the stronger predictor of MCI (adjHR 4.04; 95% CI 1.41–11.3) while the presence of MCI at baseline (adj HR 7.55; 95% CI 1.76–32.3) and a severe WMLs burden (adj HR 2.80; 95% CI 0.86–9.04) were the strongest predictors of PDD, even if this latter association has a trend towards significance.

**Conclusion:**

Hypertension represents the most important modifiable risk factor for PD-MCI in the PACOS cohort, increasing the risk of about four times.

## Introduction

Cognitive impairment, from mild cognitive impairment (MCI) to dementia (PDD), is one of the most common and disabling non-motor symptoms of Parkinson’s disease (PD) [1].

Despite the efforts to improve the knowledge pertaining the pathological bases of cognitive decline in PD, the contribution of vascular pathology is still unclear.

In the general population, some modifiable vascular risk factors (VRFs) including hypertension, diabetes, obesity and hypercholesterolemia have been associated with cognitive impairment [2, 3]. In particular, it has been suggested that VRFs may cause microvascular dysfunction leading to chronic hypoperfusion and white matter lesions (WMLs) [4]. In particular, VRFs contribute to damage neuronal circuits involved in cognition, including those connecting thalamus, basal ganglia, internal capsule and brainstem to the frontal lobes [5].

In PD, some studies have associated the co-occurrence of VRFs and WMLs with worse motor and cognitive performances, particularly in late-onset PD [6, 7]. Given that cognitive impairment represents a frequent condition even in de novo PD subjects in early stages [8, 9], the need to define whether VRFs represent a modifiable risk factor for slowing down or even prevent cognitive decline is urgent. Nevertheless, to date only few studies, three cross-sectional and three longitudinal, have investigated the effects of VRFs on PD-MCI and PDD occurrence according to the Movement Disorder Society (MDS) level II diagnostic criteria for PD-MCI [10, 11], reporting inconclusive results [7, 12–16].

This study is part of The PArkinson’s disease COgnitive impairment Study (PACOS), an observational study involving two Sicilian centers, aimed to assess frequency, clinical features and biomarkers associated with MCI in a large hospital-based cohort of patients affected by PD [8, 9, 17–19]. The aim of the present study was to evaluate the role of VRFs and WMLs burden as putative risk factors for the occurrence of PD-MCI and its progression to PDD.

## Materials and methods

### Study population

PD patients diagnosed according to the Brain Bank criteria [20] who attended the Neurologic Unit of the “Policlinico Vittorio Emanuele” in Catania and the Memory and Parkinson’s disease Center of the “Policlinico Paolo Giaccone” in Palermo, were retrospectively enrolled in the PACOS cohort. The population included 659 non-demented PD subjects at baseline. All participants underwent a standard neurological workup, including a comprehensive neuropsychological assessment. Background and methods have been extensively reported elsewhere [8, 9].

We retrospectively enrolled all PD patients who underwent at least two comprehensive neuropsychological evaluation (baseline and follow-up) between 2014 and 2017 during a period of maximum 48 months (between 12 and 48 months). All participants provided written informed consent prior to entering the study, which has been approved by the local Ethical Committee and was in accordance with the Declaration of Helsinki.

### Clinical assessment

All patients, at baseline and follow-up, underwent a standard neurological examination performed by neurologists experienced in Movement Disorders. Demographic, clinical and pharmacological data were collected from patients’ medical records. PD severity was evaluated with the Unified Parkinson Disease Rating Scale-Motor Evaluation (UPDRS-ME) and the Hoehn–Yahr (HY) scale. All motor evaluations have been conducted in “off” state. The clinical phenotype has been attributed according to the classification in Tremor Dominant (TD), Postural Instability Gait Difficulty (PIGD) and Undetermined using scores from part II and III of UPDRS [21].

### Neuropsychological and behavioral assessment

At baseline and follow-up examinations, all PD subjects underwent a comprehensive neuropsychological and behavioral assessment in “on” state. The following five cognitive domains were evaluated with two tests for domain: episodic memory (Rey’s Auditory Verbal Learning Test and Prose recall test with a delayed recall condition); attention (Stroop color-word test and Trail Making Test part A); executive functioning (Verbal fluency letter test and Colored Raven’s Progressive Matrices); visuo-spatial functioning (Clock drawing test and Copy of figures); language (Aachener Aphasie Test-Naming item and the short version of the Token test). Neuropsychological tests were considered as “impaired” when the subject scored two standard deviation (SD) below normality cut-off values. Diagnosis of PD-MCI was made according to the Movement Disorder Society Task Force criteria-level II [10]. Diagnosis of PDD was made according to the MDS criteria [11]. Details about the neuropsychological assessment used in the PACOS have been extensively reported elsewhere [8, 9].

### Assessment of VRFs

Vascular risk factors, comorbidities and medications were evaluated at baseline. Data have been recorded from clinical record of patients. Vascular comorbidities included history of diabetes, hypertension and the use of anti-hypertensive drugs, hypercholesterolemia and hypertriglyceridemia. History of myocardial infarction, coronary artery bypass graft, angioplasty or stenting, atrial fibrillation, or valvulopathy determined a diagnosis of heart disease. History of TIA or stroke as well as tobacco use, has been also collected. Serum lipids (LDL/HDL/total cholesterol), triglycerides and glycaemia have been extracted from clinical records. Systolic and diastolic pressures have been recorded from the cardiologic examination during patient admission and as the result of three different measurements three minutes apart.

To assess the role of VRFs on cognition, patients were classified according to the simplified 10-year Framingham General Cardiovascular Disease Risk Score [22]. The ratio between individual FR score and normative risk score has been used to classify patients in High Vascular Risk (HVR; ratio > 1.0) and Low Vascular Risk (LVR; ratio ≤ 1.0) [13].

### White matter lesions assessment

Patients underwent 1.5 T MRI scan (Signa HDxt, GE Medical Systems, Milwaukee, WI, USA) ± 1 month the baseline neuropsychological evaluation.

WMLs were defined as areas of hyperintensities on either T2 or FLAIR images, using a validated semiquantitative method proposed by Wahlund et coll [23]. Two blinded neurologists have independently evaluated the findings on MRI images.

### Statistical analysis

Data were analyzed using STATA 12.1 software packages (StataCorp, College Station, TX, USA). Quantitative variables were described using mean and standard deviation. The difference between means and proportions was evaluated by the t-test and the Chi square test, respectively. In case of a not normal distribution, appropriate non-parametric tests were performed.

For incident PDD cases, we assigned time of dementia onset to the midpoint of the interval between assessments at which dementia was diagnosed. Because PD-MCI, in contrast to PDD, may be reversible or fluctuate over time, we set time of onset of incident PD-MCI to the exact date at which PD-MCI was first diagnosed. To identify possible predictors of progression from NC to MCI or PDD among the clinical and demographic characteristics, Cox proportional-hazards regression model was used for both the univariate and multivariate analyses. Variables with *p* value < 0.1 at univariate analysis were included in the final multivariate Cox models. Age, sex, UPDRS-ME and education were considered a priori confounders and included in the model regardless the significance level. Schoenfeld residuals test was used for testing the proportional hazard. 95% confidence interval (CI), and *p* value (two-tailed test, *a* = 0.05).

Whenever variables were dichotomized or polychotomized, the cut-offs were derived from the pooled distribution of cases and control subjects (e.g., using the median value). Levodopa equivalent daily dose (LED) was calculated [24].

The Wahlund score was considered as the total score (range 0–30) and was also polychotomized into three levels according to the pooled distribution (level 0 = absence of lesions; level 1 when the total score was between 1 and 4; level 2 when the total score was > 4).

## Results

The PACOS cohort consists of 659 non-demented PD patients [8]. Of the 659 subjects, 139 PD patients (men 87, 62.6%) with a mean disease duration of 3.0–2.8 years who underwent at least two neuropsychological evaluations between 12 and 48 months from 2014 to 2017 were enrolled in the present study. When compared to the baseline cohort, patients enrolled in this study were comparable in terms of demographic and clinical characteristics, except for a slightly shorter disease duration [9].

Of the 139 patients at baseline (first neuropsychological evaluation), 84 (60.4%) were classified as PD with normal cognition (PD-NC), while 55 (39.6%) fulfilled the diagnosis of PD-MCI. Baseline characteristics are reported in Table 1.

**Table 1:** Demographic and clinical characteristics at baseline

	NC (*N* = 84)	MCI (*N* = 55)	Total (*N* = 139)	*p* value
Men	52 (61.9)	35 (63.6)	87 (62.6)	0.8
Age, years	64.4 ± 10.4	67.5 ± 7.4	65.7 ± 9.4	0.07
Age at onset, years	61.6 ± 11.0	64.5 ± 7.8	62.8 ± 10.0	0.09
Education, years	9.3 ± 4.4	8.3 ± 4.6	8.9 ± 4.6	0.2
UPDRS-ME score	25.4 ± 14.5	27.4 ± 11.9	26.2 ± 13.5	0.4
HY stage	1.9 ± 0.6	2.2 ± 0.7	2.0 ± 0.7	**0.02**
Disease duration, y	3.0 ± 2.9	3.0 ± 2.7	3.0 ± 2.8	0.9
Depression	29 (34.5)	22 (40.0)	51 (36.7)	0.4
LED mg/day	437.2 ± 463.8	397.9 ± 408.8	421.8 ± 442.0	0.6
Phenotype				
TD	32 (38.1)	11 (20.0)	43 (30.9)	/
PIGD	47 (55.9)	39 (70.9)	86 (61.9)	/
Mixed	5 (5.9)	5 (9.1)	10 (7.2)	0.07

Data presented are number (percentage %) for categorical and mean ± SD for continuous data. Significant results are expressed in bold

*NC* normal cognition, *MCI* mild cognitive impairment, *UPDRS-ME* Unified Parkinson’s Disease Rating Scale-Motor Examination, *HY* Hoeh-Yahr, *LED* levodopa equivalent daily dose, *TD* tremor dominant, *PIGD* postural instability gait difficulty

Considering the whole sample of 139 enrolled PD patients, patients with a diagnosis of MCI at baseline were more likely to have reported a history of diabetes, hypertension, use of anti-hypertensive drugs and had a higher baseline both systolic (SBP) and diastolic blood pressure (DBP) as shown in Table 2.

**Table 2:** Vascular risk factors at baseline

Vascular risk factors	NC (*N* = 84)	MCI (*N* = 55)	*p* value
Diabetes (H)	10 (12.2)	16 (29.1)	**0.01**
Hypercholesterolemia (H)	26 (31.7)	20 (36.3)	0.50
Hypertension (H)	46 (56.1%)	40 (72.73)	**0.03**
Hypertrigliceridemia (H)	9 (11.0)	11 (20.0)	0.12
SBP mmHg	127.5 ± 8.3	133.8 ± 16.9	**0.01**
DBP mmHg	76.0 ± 7.2	80.4 ± 10.0	**0.01**
SBP > 130 (median value)	30 (35.7)	27 (49.1)	0.1
SBP > 140	12 (14.3)	13 (23.6)	0.2
Antihypertensive drugs	44 (52.4)	39 (70.9)	**0.03**
HDL mg/dl	48.0 ± 11.8	49.5 ± 9.7	0.41
Total cholesterol mg/dl	180.2 ± 36.1	177.4 ± 32.7	0.6
Smoking	15 (19.7)	11 (20.3)	0.3
Cigarette/die^a^	17.8 ± 11.7	16.6 ± 13.4	0.99
Atrial fibrillation	1 (1.2)	3(5.4)	0.1
FR Score (HVR)	58(69.0)	42 (75.4)	0.3
Myocardial infarction	9 (11.0)	6 (10.9)	0.9
Stroke	0	1(1.8)	/
TIA	0	0	/
Average Wahlund	2.2 ± 2.8	3.3 ± 4.5	**0.07**
Wahlund score			
0 (score = 0)	39 (46.4)	26 (47.3)	
1 (score 1–4)	34(40.2)	9 (16.4)	
2 (score > 4)	11 (13.1)	20 (36.4)	

Data presented are number (percentage %) for categorical and mean ± SD for continuous data. Significant results are expressed in bold

*NC* normal cognition, *MCI* mild cognitive impairment, *H* history, *SBP* systolic blood pressure, *DBP* diastolic blood pressure, *HDL* high density lipoproteins, *FR score HVR* Framingam Risk Score, high vascular risk, *TIA* transient ischemic attack

^a^Number of cigarette/day was available for 126 subjects

At baseline according to the FR score, 39 patients (28.1%) were classified as LVR, while 100 (71.9%) as HVR. HVR was more frequent among the 55 PD-MCI at baseline with respect to the PD-NC (76.4% versus 69.0%), but such difference was not statistically significant (*p* value 0.3).

### Vascular risk factors and risk of PD-MCI

**Fig. 1:**
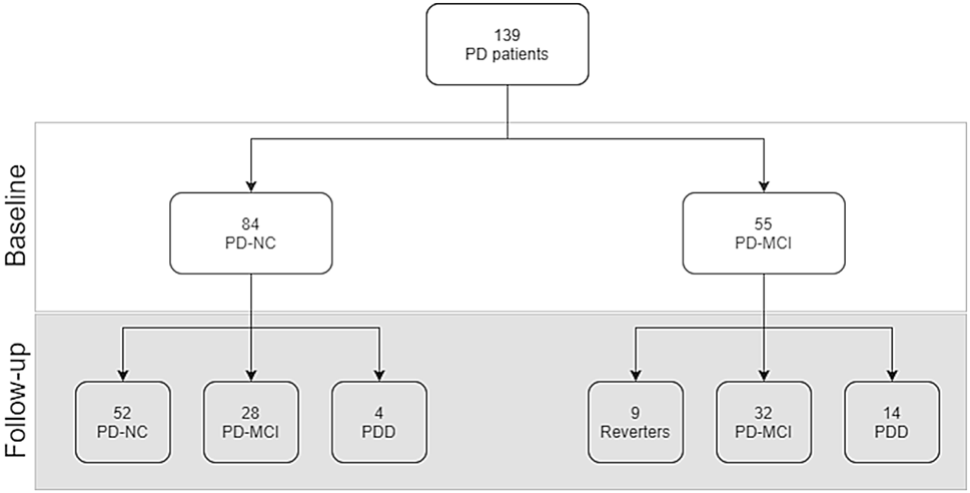
Flowchart of the evolution of PD-NC and PD-MCI from baseline to follow-up. PD Parkinson’s disease, NC normal cognition, *PDD* Parkinson’s disease dementia

Considering the 84 PD-NC at baseline, 28 (33.3%) fulfilled the diagnosis of PD-MCI, while 4 (4.8%) fulfilled the diagnosis of PDD at follow-up (mean follow-up time 23.5 ± 10.3 months) as displayed in Fig. 1.

At univariate analysis age at baseline and use of antihypertensive drugs were positively associated with the risk of MCI. As previously reported, education was negatively associated with the development of MCI [8, 9].

Considering the presence of hypertension, at univariate analysis, we found a positive association for history of hypertension with a trend towards significant. Furthermore, an association with a trend towards significant was also found for the presence of a SBP > 130 mmHg (median value of the pooled distribution), as well as with SBP > 140 mmHg, dichotomized according to the current definition of hypertension [25]. The presence of a Wahlund score greater than four was associated with the risk of MCI with a trend towards significance, as shown in Table 3.

**Table 3:** Development of PD-MCI considering the 84 PD-NC at baseline. Cox proportional-hazards regression models

	MCI *N* = 28	No-MCI *N* = 52	Univariate analysis				Multivariate analysis		
			HR	95% CI		*p* value	HR	95% CI	*p* value
Men	18 (64.3)	33 (63.4)	1.30	0.58–2.90		0.6	0.66	0.25–1.70	0.4
Age, years	68.5 ± 9.8	62.2 ± 10.3	1.04	1.00–1.09		**0.04**	1.63	0.99–1.10	0.1
Age at onset, years	65.2 ± 10.8	59.6 ± 11.0	1.04	1.00–1.08		**0.06**			
UPDRS-ME	24.4 ± 13.0	24.6 ± 14.2	0.98	0.95–1.00		0.2	0.97	0.94–1..00	**0.05**
HY stage	2.1 ± 0.9	1.9 ± 0.5	1.21	0.76–1.94		0.4			
Disease duration, y	3.3 ± 3.1	2.6 ± 2.7	1.01	0.90–1.14		0.8			
Education, years	7.5 ± 4.8	10.5 ± 4.1	0.90	0.82–0.98		**0.02**	0.90	0.82–0.98	**0.02**
* Education* ≤ 8	21 (75.0)	22 (42.3)	1	/		**/**			
* Education* > 8	7 (25.0)	30 (57.7)	0.35	0.15–0.82		**0.02**			
LED mg/day	480.8 ± 569.5	425.0 ± 415.6	1.00	0.99–1.00		0.5			
Depression	13 (46.4)	45 (31.8)	1.80	0.83–3.88		0.1			
Phenotype									
TD	11 (39.3)	19 (42.2)	1						
PIGD	15 (53.6)	24 (53.3)	0.75	0.34–1.65		0.5			
Mixed	2 (7.1)	2 (4.4)	3.50	0.72–16.9		0.1			
Smoking	4 (36.4)	11 (18.9)	0.78	0.27–2.32		0.7			
Diabetes (H)	6 (21.4)	4 (7.7)	1.12	0.45–2.78		0.8			
Hypercholesterolemia (H)	7 (25.0)	17 (32.7)	0.65	0.28–1.54		0.3			
Hypertension (H)	6 (21.4)	3 (5.7)	2.16	0.94–4.94		0.07			
Hypertrigliceridemia (H)	19 (67.8)	24 (46.1)	1.53	0.61- 3.81		0.4			
SBP, mmHg	132.1 ± 12.2	125.5 ± 13	1.02	0.99 -1.05		0.2			
DBP, mmHg	77.6 ± 8.2	75.2 ± 6.5	1.01	0.97- 1.06		0.6			
SBP > 130 *(median value)*	14 (50.0)	14 (26.9)	1.93	0.92- 4.05		**0.08**			
SBP > 140	7 (25.0)	4 (7.7)	1.78	0.75–4.21		**0.1**	4.04	1.41–11.3	**0.009**
Antihypertensive drugs	20	21	2.85	1.25- 6.52		**0.01**			
HDL, mg/dl	50.0 ± 13.4	46.2 ± 10.0	1.01	0.98- 1.04		0.4			
Total cholesterol, md/dl	175.2 ± 31.8	180.6 ± 38.3	1.00	0.99–1.01		0.9			
Atrial Fibrillation	1 (3.57)	0	/	/		/			
FR Score (HVR)	21 (75)	34 (65.4)							
Myocardial Infarction	3 (10.7)	6 (11.5)	0.57	0.17- 1.90		0.4			
Stroke	0	0	/	/		/			
TIA	0	0	/	/		/			
Average Wahlund	1.7 ± 2.3	3.0 ± 3.5	1.07	0.96–1.20		0.2			
Wahlund score									
0 (score = 0)	13 (46.4)	25 (48.1)	1	/		/			
1 (score 1–4)	7 (25)	24 (46.1)	0.79	0.31–1.98		0.6			
2 ( score > 4)	8 (28.6)	3 (5.8)	2.17	0.89–5.29		**0.09**			

Data presented are number (percentage %) for categorical and mean ± SD for continuous data. Significant results are expressed in bold

*MCI* mild cognitive impairment, *NC* normal cognition, *HR* hazard ratio, *UPDRS-ME* Unified Parkinson’s Disease Rating Scale-Motor Examination, *HY* Hoeh-Yahr, *LED* levodopa equivalent daily dose, *TD* tremor dominant, *PIGD* postural instability gait difficulty, *H* history, *SBP* systolic blood pressure, *DBP* diastolic blood pressure, *HDL* high density lipoproteins, *FR score HVR* Framingam Risk Score, high vascular risk, *TIA* transient ischemic attack

On the other hand, diabetes, hypercholesterolemia, hypertriglyceridemia, atrial fibrillation, TIA or stroke as well as FR score were not associated with the risk of MCI**.**

At multivariate analysis, adjusting by age, sex, education and UPDRS-ME, we found an association with a trend towards significance between the use of antihypertensive drug and the risk of MCI (HR 2.32; 95% CI 0.95–5.66; *p* value 0.06). However, when the multivariate model was built considering the presence of a SBP > 140 a four times increased risk of MCI was recorded (HR 4.04; 95% CI 1.41–11.3; *p* value 0.009) as shown in Table 3. A close HR was found when the analysis was adjusted by the use of antihypertensive drugs (HR 3.37; 95% CI 1.48–8.91; *p* value 0.03). It should be noted that, a more than 3-times increased risk of MCI was also recorded also considering a SBP > 130 mmHg, adjusting by age, sex education and UPDRS-ME (HR 3.59, 95% CI 1.47–8.74; *p* value 0.005). DBP was not associated with PD-MCI either at univariate or multivariate analysis and did not modify the strength of association of SBP. No significant association was found at multivariate analysis between presence of WMLs and risk of MCI. Multivariate analysis is reported in Table 3.

### Vascular risk factors and risk of PDD

Considering the entire sample of 139 PD patients, 18 fulfilled the diagnosis of PDD at follow-up (mean follow-up time 24.0 ± 10.2 months) (Fig. 1). At univariate analysis age at baseline and education were significantly associated with the risk of developing dementia. Presence of MCI at baseline was the strongest predictor of PDD (HR 4.37; 95% CI 1.42–13.5, *p* value 0.01) along with the presence of WMLs at baseline (Wahlund score > 4 h 3.81, 95% CI 1.28–11.3; *p* value 0.01). None of the vascular risk factors evaluated were associated with the development of PDD including the FR score. At multivariate analysis, adjusting by age, sex, education and UPDRS-ME, the presence of MCI remained the strongest predictor of PDD (HR 7.55; 95% CI 1.76–32.3; *p* value 0.006) along with male sex (HR 4.94; 95% CI 1.23–18.80; *p* value 0.02). Presence of WMLs increased the risk of PDD of almost 3-times, with an association with a trend towards significance (Wahlund score > 4 h 2.80; 95% CI 0.86–9.04; *p* value 0.08) (Table 4).

**Table 4:** Development of PDD. Cox proportional-hazards regression models

	PDD *N* = 18	No-PDD *N* = 121	Univariate analysis			Multivariate analysis		
			HR	95% CI	*p* value	HR	95% CI	*p* value
Men	12 (66.7)	75 (62.0)	1.21	0.44–3.37	0.7	4.94	1.23–18.8	**0.02**
Age, years	68.3 ± 8.4	65.3 ± 9.5	1.07	1.00–1.15	**0.04**	1.05	1.00–1.14	0.2
Age at onset, years	64.9 ± 8.4	62.4 ± 10.1	1.05	0.99–1.12	0.1			
UPDRS-ME	33.1 ± 16.5	25.1 ± 12.7	1.03	1.00–1.05	0.06	1.03	1.00–1.07	**0.05**
HY stage	1.9 ± 0.6	2.1 ± 0.7	0.77	0.40–1.47	0.4			
Disease duration, years	3.4 ± 2.8	2.9 ± 2.8	1.07	0.92–1.25	0.3			
Education, years	7.4 ± 4.8	9.2 ± 4.5	0.88	0.78–0.99	**0.04**	0.83	1.29–18.7	**0.03**
LED mg/day	353.8 ± 298.4	432.1 ± 459.8	1.00	1.00–1.001	0.9			
Cognition baseline								
NC	4 (22.2)	80 (66.1)	1	/	/			
MCI	14 (77.8)	41 (33.9)	4.37	1.42–13.5	**0.01**	7.55	1.76–32.3	**0.006**
Depression	9 (50.0)	42 (34.7)	1.28	0.50–3.24	0.6			
Phenotype								
TD	6 (33.3)	37 (30.6)	1	/	/			
PIGD	12 (66.7)	74 (61.2)	1.01	0.38–2.76	0.9			
Mixed	0	10 (8.3)	/	/	/			
Smoking	2 (12.5)	24 (21.0)	0.52	0.11–2.36	0.4			
Diabetes (H)	3 (16.7)	23 (19.0)	0.75	0.21–2.68	**0.7**			
Hypercholesterolemia (H)	5 (27.8)	41 (33.9)	0.66	0.23–1.88	0.4			
Hypertension (H)	14 (77.8)	72 (59.5)	2.09	0.68–6.44	0.2			
Hypertrigliceridemia (H)	2 (11.1)	18 (14.9)	0.75	0.16–3.30	0.7			
SBP, mmHg	129.2 ± 19.0	130.1 ± 14.8	1.00	0.97–1.03	0.9			
DBP, mmHg	80 ± 11.5	77.4 ± 8.2	1.03	0.99–1.09	0.1			
SBP > 130 (median value)	10 (55.6)	47 (38.8)	1.89	0.70–5.02	0.2			
SBP > 140	4 (22.2)	21 (17.4)	1.17	0.38–3.61	0.8			
Antihypertensive drugs	14 (77.8)	69 (57.0)	2,30	0.74–7.05	0.1			
HDL, mg/dl	48.4 ± 12.4	48.6 ± 10.8	0.99	0.94–1.03	0.7			
Total cholesterol, mg/dl	182 ± 38.0	178.7 ± 34.4	1.00	0.99–1.01	0.6			
Atrial Fibrillation	1 (5.6)	3 (2.5)	2.24	0.29–17.2	0.4			
FR Score (HVR)	14 (77.8)	86 (71.1)	1.24	0.40–3.81	0.7			
Myocardial Infarction	1 (5.6)	14 (11.6)	0.44	0.05–3.38	0.4			
Stroke	/	1 (0.8)	/	/	/			
TIA	/	/	/	/	/			
Average Wahlund	4.3 ± 4.3	2.4 ± 3.3	1.16	1.04–1.30	**0.007**			
Wahlund score								
0 (score = 0)	6 (33.3)	59 (48.8)	1	/	/	1	/	/
1 (score 1–4)	4 (22.2)	39 (32.2)	0.71	0.16–3.02	0.6	2.31	0.47–11.2	0.3
2 (score > 4)	8(44.4)	23 (19.0)	3.81	1.28–11.3	**0.01**	2.80	0.86–9.04	**0.08**

Data presented are number (percentage %) for categorical and mean ± SD for continuous data. Significant results are expressed in bold

*PDD* Parkinson’s disease dementia, *HR* hazard ratio, *UPDRS-ME* Unified Parkinson’s Disease Rating Scale Motor Examination, *HY* Hoeh-Yahr, *LED* levodopa equivalent daily dose, *NC* normal cognition, *MCI* mild cognitive impairment, *TD* Tremor Dominant, *PIGD* postural instability gait difficulty, *H* history, *SBP* systolic blood pressure, *DBP* diastolic blood pressure, *HDL* high density lipoproteins, *FR score HVR* Framingam Risk Score, high vascular risk, *TIA* transient ischemic attack

However, it should be noted that when Wahlund score was included in the model as continuous variable the association was significant (HR 1.15; 95% CI 1.01–1.31; *p* value 0.02) and a close HR was found for MCI at baseline (HR 6.62; 95% CI 1.87–23.5; *p* value 0.003) and male sex (HR 5.00; 95% CI 1.34–18.62; *p* value 0.02).

## Discussion

The specific contribution of vascular pathology in PD cognitive dysfunction is still debated. Several studies that have evaluated the possible role of VRFs and WMLs were performed before the new PD-MCI criteria and, for the different definitions adopted, results are scarcely comparable [26, 27].

To date, few cross-sectional [7, 12, 13] and longitudinal studies [14–16] have evaluated the role of both VRFs and WMLs in the risk of developing cognitive decline in PD patients using the new MDS criteria but conflicting results have been reported also by these more recent studies. In particular, different study design (cross-sectional versus longitudinal), different levels of PD-MCI MDS criteria (level I versus level II), different measures or definition of VRFs (Framingham Index rather than singular VRFs; self-reported history versus instrumental evaluation of VRFs), different estimation of WMLs (visual versus automatic) as well as the different MRI equipment can largely affect the results.

### VRFs and WMLs and risk of PD-MCI

Out of the 84 PD-NC at baseline, 33.3% developed MCI at follow-up. As already reported elsewhere, older age and low education level have been significantly associated with the risk of MCI occurrence [9]. Considering the VRFs in the present study, hypertension was the most important predictor of MCI at multivariate analysis. In particular, a 4-times increased risk of PD-MCI has been recorded among patients with SBP > 140 mmHg and a 3-times increased risk has been also recorded for PD patients with SBP > 130 mmHg. A similar result was found when antihypertensive drugs were used as surrogate marker for hypertension, thus confirming its role regardless the different types of measurement. This finding is in agreement with the longitudinal study carried out by Park et al. were hypertension was strongly associated with the conversion from PD-NC to PD-MCI [16]. Also in the cross-sectional study carried out by Pilotto et al. hypertension was significantly more frequent in PD-MCI and dementia subgroups, even if this association was not significant at multivariate analysis [12]. However, neither the cross-sectional study carried out by Malek et al. [7] nor the longitudinal one performed by Sunwoo et al. [15] reported an association between hypertension and PD-MCI. In some studies, the possible role of VRFs was evaluated only considering the FR score [13, 14]. We did not find any association between FR score and PD-MCI and this result is agreement with the Parkinson’s Progression Markers Initiative study [14]. Contrarily, in the cross-sectional study recently carried out by Stojkovic and coll, the FR score was associated with PD-MCI [13].

Concerning WMLs, we found an association with a trend towards significance with a Wahlund score greater than 4 (HR 2.17; *p* value 0.09) and PD-MCI. This finding is in agreement with the longitudinal studies carried out by Chahine et al. [14] and by Sunwoo et al. [15] Similarly, Stojkovic et al. reported an association between WMLs and PD-MCI with a trend towards significance [13]. However it should be noted that in the study carried out by Park et al. [16] the presence of severe cerebral small vessel disease in the basal ganglia was found to be strongly associated with PD-MCI.

In our study we did not find any association between tobacco smoking and occurrence of neither PD-MCI nor PDD. In the general population, the role of nicotine in favoring or preventing cognitive decline is still controversial. In particular, if on the one hand nicotine increase oxidative stress-related cerebrovascular damage, favoring cognitive decline, on the other hand it could counterbalance cholinergic deficits preventing dementia.[27]. However, a recent meta-analysis of prospective cohort study, reported that tobacco smoking increased the risk of cognitive decline in PD [28].

### VRFs and WMLs and the risk of PDD

Considering the 139 non-demented patients at baseline, 12.9% developed PDD at follow-up. As reported elsewhere, MCI was the stronger predictor of PDD along with male sex while education was protective [9].

Along with these recognized risk factors, PDD was strongly predicted by the WMLs burden at baseline. At multivariate analysis, an almost 3-time risk was found for a Wahlund score > 4 even if such association was with a trend towards significance (HR 2.80; *p* value 0.08). Nonetheless, it should be noted that the association became significant when Wahlund score was included in the model as continuous variable (HR 1.15; *p* value 0.02). This finding is in agreement with the studies carried out by Stojkovic, Sunwoo and Park [13, 15, 16] which showed that the presence of WMLs was not associated with PD-MCI but only with PDD. Conversely, none of the VRFs evaluated was significantly associated with PDD.

Hypertension is a recognized risk factor for cognitive decline in PD [29–32]. However, in the present study, the presence of hypertension at baseline was only associated with the development of MCI and not of PDD, the latter associated only with the WMLs burden.

MCI is present in about 30% of incident PD patients and represents the most important risk factors for the development of PDD [8, 9]. Hypertension, especially if uncontrolled, is a major risk factor for the development of WMLs, leading to vascular cognitive impairment [31]. Hypertension, in fact, leads to structural changes of cerebral vessels as adaptive consequence to counteract the increased transmural pressure. However, over the time, these structural changes predispose to different pathologies, including microatheroma, microinfarcts, microbleeds, lacunae, atherosclerosis, resulting in WMLs [33]. Moreover, it has been reported that patients suffering from hypertension, frequently experience orthostatic hypotension, the latter in turn associated with both cognitive decline [34, 35] and cerebral hypoperfusion [36].

Hence, it could be hypothesized that, in an early stage hypertension but not WMLs, increases the risk of MCI. Subsequently, while the neuropathological vascular damage driven by hypertension prosecutes, the development of WMLs, an irreversible factor, contributes to PDD occurrence. Thus, the maintenance of an appropriate blood pressure control, could represent a feasible strategy to reduce MCI, WMLs and, finally, PDD occurrence.

Our study has several strengths, including the large PACOS cohort size at baseline [8], the application of MDS Level II diagnostic criteria for PD-MCI diagnosis and the assessment of VRFs with different sources to evaluate the presence of different exposures (e.g. self-reported history of hypertension, use of antihypertensive drugs and blood pressure measurement; self-reported history of hypercholesterolemia, serum lipid levels, etc.). To the best of our knowledge, this is the largest longitudinal study which simultaneously assessed the combined role of several VRFs and WMLs in subjects fulfilling the MDS Level II requirements for *PD*-MCI conducted to date.

Nonetheless, several limits should be considered in interpreting our data. First, a possible selection bias cannot be excluded due to the hospital-based study design. As for other hospital-based cohorts, presence of more severe cases cannot be excluded, and this may possibly have contributed to the high estimate of MCI at baseline. Nonetheless the average HY score and the short disease duration recorded in the PACOS cohort have revealed a mild to moderate stage of disease [8, 9]. Furthermore, although analyses were adjusted for major potential confounders, residual confounding cannot be excluded. A further limit is related to the evaluation of the WMLs burden that was estimated with the visual score of Wahlund and not with a software package. However, it should be noted that the scoring was performed by two neurologists blinded to clinical information of patients. Finally, probably due to the relatively small sample of PD-NC that develop PD-MCI at follow-up, we obtained wide CIs thus limiting the generalizability of our results.

In conclusion, results of the present report suggest that hypertension is the strongest vascular risk factor for the development of cognitive impairment in PD. Considering that hypertension is a modifiable risk factor, its control might have a role in preventing PD-MCI and, consequently, PDD occurrence. Thus, this data have relevant prognostic and therapeutic implications. Larger prospective cohort studies, as well as blood pressure intervention trials are needed to confirm the role of hypertension as modifiable risk factor for cognitive impairment in PD.

## Data Availability

Anonymized data are available if request.
